# Probable Ankylosaur Ossicles from the Middle Cenomanian Dunvegan Formation of Northwestern Alberta, Canada

**DOI:** 10.1371/journal.pone.0096075

**Published:** 2014-05-09

**Authors:** Michael E. Burns, Matthew J. Vavrek

**Affiliations:** 1 Biological Sciences, University of Alberta, Edmonton, Alberta, Canada; 2 Pipestone Creek Dinosaur Initiative, Clairmont, Alberta, Canada; University of Pennsylvania, United States of America

## Abstract

A sample of six probable fragmentary ankylosaur ossicles, collected from Cenomanian deposits of the Dunvegan Formation along the Peace River, represent one of the first dinosaurian skeletal fossils reported from pre-Santonian deposits in Alberta. Specimens were identified as ankylosaur by means of a palaeohistological analysis. The primary tissue is composed of zonal interwoven structural fibre bundles with irregularly-shaped lacunae, unlike the elongate lacunae of the secondary lamellar bone. The locality represents the most northerly Cenomanian occurrence of ankylosaur skeletal remains. Further fieldwork in under-examined areas of the province carries potential for additional finds.

## Introduction

Although Alberta is one of the most intensely studied areas in the world in regards to dinosaur palaeontology [1]–[2], skeletal fossils of dinosaurs from pre-Santonian rocks are virtually unknown [3]. Other than a recently recovered ankylosaur from marine sediments of the Albian Clearwater Formation of northeastern Alberta [4], and an isolated *Ichthyornis* sp. humerus from the Turonian Kaskapau Formation [5], no other dinosaur skeletal remains have been described from Alberta in rocks older than Santonian, although there does exist a non-descriptive reference to Dunvegan Formation dinosaurs in an encyclopedia article [6]. During the course of research for this paper, the authors were informed of some undescribed, indeterminate bones (possibly ornithischian) from the Blairmore Formation of southwestern Alberta (D. Brinkman, pers. comm.), however this material is highly fragmentary.

Even outside of Alberta, there are virtually no skeletal remains of pre-Santonian dinosaurs from north of the 49^th^ Parallel. There are records of two possible ornithischian vertebrae and several birds from the from the Belle Fourche Member of the Ashville Formation in eastern Saskatchewan [7]–[8], undescribed dinosaur bones from the Turonian Kaskapau Formation of British Columbia [9], an indeterminate hadrosaur from the Turonian Matanuska Formation of Alaska [10]–[11], and a possible bone fragment from the Late Jurassic of Alaska [12]. Despite the lack of a recognized fossil record, there are relatively large exposures of lower Upper Cretaceous rocks in Alberta, primarily concentrated along large river channels in the northern portion of the province.

During a survey of the Cenomanian-aged Dunvegan Formation along the Peace River, a handful of vertebrate remains were recovered, including six probable fragmentary ankylosaur ossicles. Although the fossils, in terms of preservational quality and completeness, are limited relative to some other dinosaur finds in western Canada, they represent an important stratigraphic and biogeographic data point because they come from an age that is poorly known in Canada, and from a location at least a thousand kilometres away from the next nearest contemporaneous skeletal record of dinosaurs.

### Geological and Climatic Setting

The Dunvegan Formation represents a middle Cenomanian-aged delta complex that occurs primarily in northeastern British Columbia and northwestern Alberta, with small extensions into the Northwest Territories [13]–[16]. The formation consists of a repeated succession of alluvial and shallow marine sandstones, siltstones and shales, and ranges between 90 and 270 m in thickness [14]–[15]. During deposition, the delta complex prograded a maximum of 400 km into the Western Interior Seaway [14]–[15]; in general, the formation becomes more terrestrial moving towards the west. The Dunvegan Formation is underlain by the marine shales of the Shaftesbury Formation, and is overlain by the marine sandstones and shales of the Kaskapau Formation [13]–[14].

**Figure 1:**
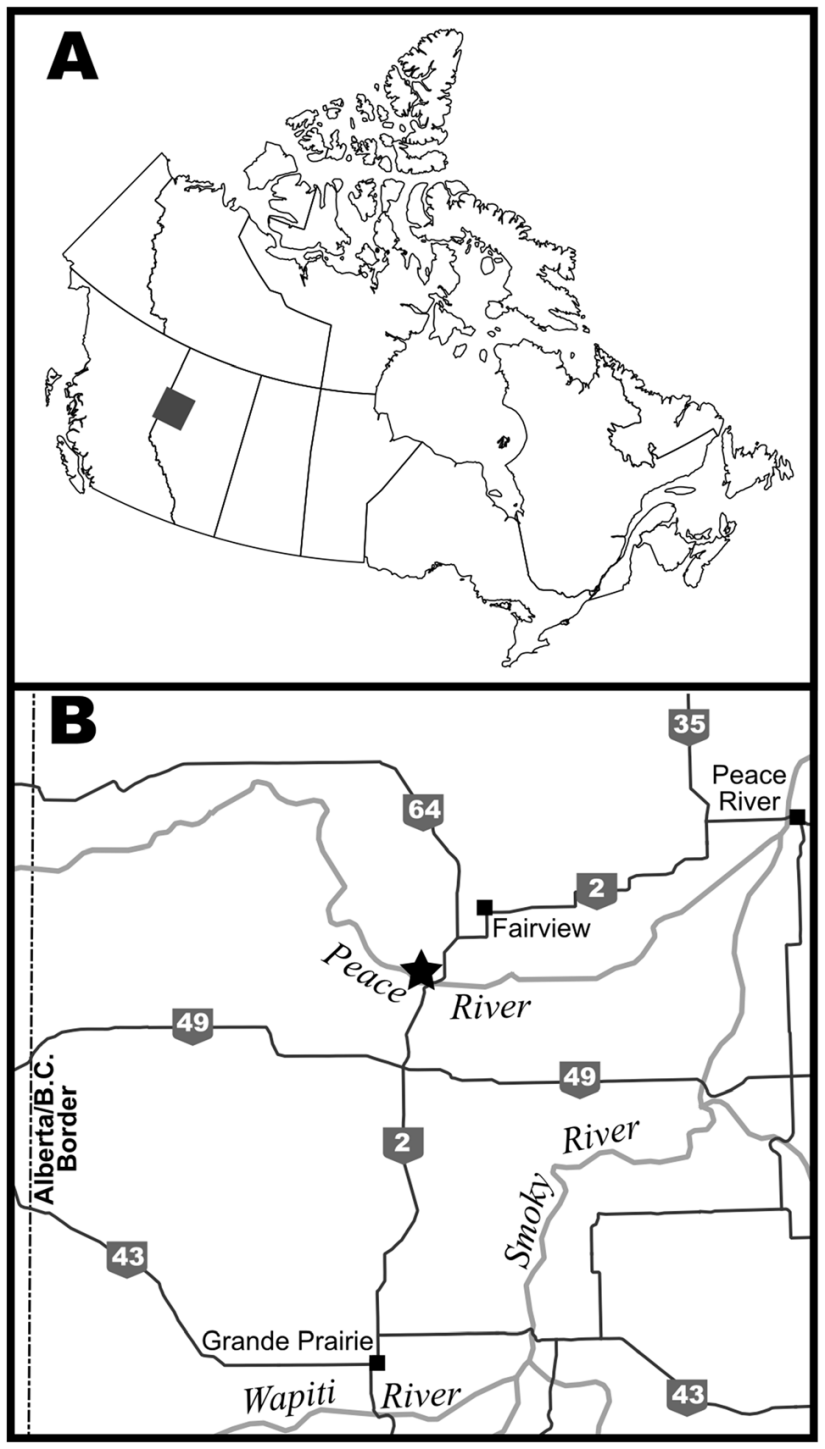
Locality map. A) Overview map showing location of inset map (B). B) Map of the Peace Country with major rivers and towns indicated. The fossil locality is denoted by a black star.

Locally, the fossils were found weathered out of a siltstone to fine sandstone bed near the top of the Peace River valley, approximately 4 km upstream of the Dunvegan Bridge (Fig. 1). The exact source of the fossils could not be determined. However, because the fossils were found near the top of an exposure, they were likely close to their original level. They were found in association with numerous ironstone pebbles that had likely weathered out from either the same beds or beds that were in close proximity.

During the Cenomanian, the Dunvegan delta was located near the Arctic Circle, at about 65° N [14]–[17] Land temperatures during this time were, however, much warmer on average than the present day [18] with higher latitude regions in particular experiencing a greater amount of warming compared to more equatorial areas, leading to a reduced equator to pole thermal gradient [19]. A study of a terrestrial ecosystem from the younger Kaskapau Formation from about the same latitude as the Dunvegan Formation suggests mean annual temperatures were around 14°C, with a cold month mean likely warmer than 5.5 C [9]. These temperatures may be high as global temperatures were on a warming trend through the Cenomanian into the Turonian [20]–[21], although the difference was likely not considerable.

## Materials and Methods

All ankylosaur skeletal specimens in this study were collected under permits obtained from Alberta Culture (Alberta Palaeontological Permit No. 12-029) and are catalogued as TMP (Royal Tyrrell Museum of Palaeontology, Drumheller, Alberta, Canada) 2012.054.0002. Photographs of ossicles were taken on a Zeiss SteREO Discovery.V8 with a Plan Apo S 0.63× objective and an attached Nikon DXM 1200C camera using NIS-Elements F 2.20 SP3 (Build 244) imaging software. To confirm its identification, one ossicle was selected for paleohistological analysis. It was stabilized via resin impregnation using Buehler EpoThin Low Viscosity Resin and Hardener. A thin section was prepared petrographically to a thickness of 100 µm and polished to a high gloss using CeO_2_ powder. The section was examined and photographed on a Nikon Eclipse E600POL trinocular polarizing microscope with an attached Nikon DXM 1200F digital camera. A composite image was constructed in Adobe Photoshop CS6 v. 13.0.1×64.

Descriptive terminology for ossicles follows [14]–[18]. Palaeohistological terminology for osteoderms follows [22]–[24]. The definition for “ossicles” adopted here is modified [25]: small (<70 mm), amorphous mineralized dermal elements often found interstitial to major osteodermal elements. The term interwoven structural fiber bundles (ISFB; sensu [23]–[26] is used to refer to mineralized metaplastic tissue dominated by large, structural collagen fibers.

## Results

**Figure 2:**
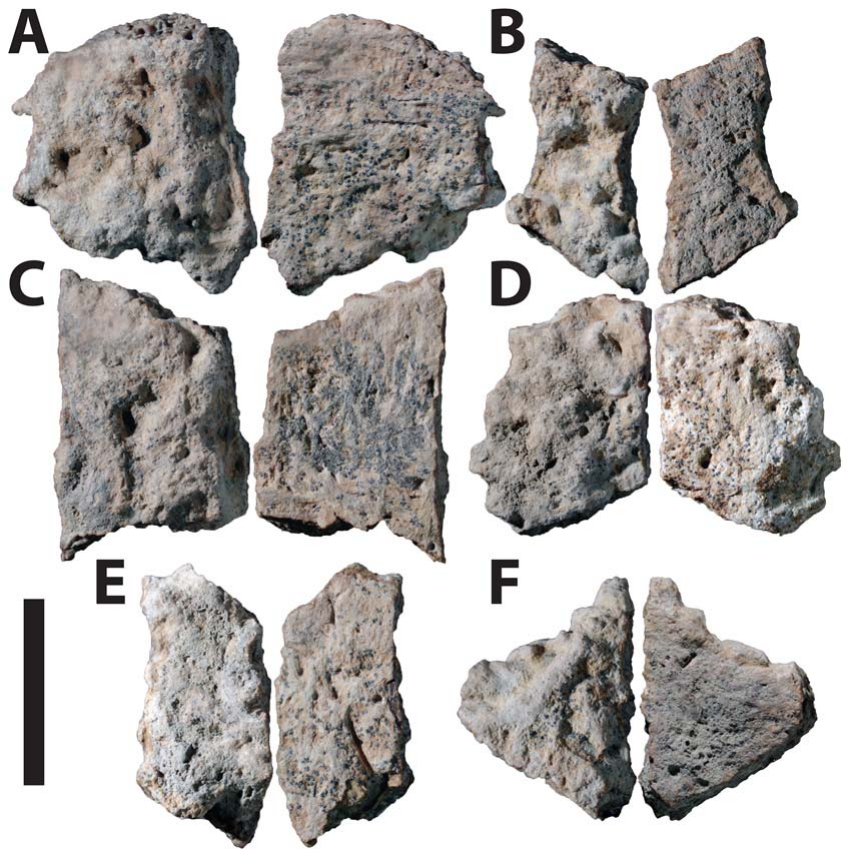
Dunvegan ossicle morphology. Ankylosaur ossicles (TMP.2012.054.0002) from the Cenomanian Dunvegan Formation, near the Peace River, British Columbia, Canada, in external and basal views. Scale bar equals 1.0 mm.

The ossicles (Fig. 2) are all irregular in shape and some are fragmentary, so it is unknown exactly how many ossicles are represented (some may be fragments of larger osteoderms). Although their exact in vivo orientation is difficult to discern, the external and basal surfaces are distinguishable. The external surfaces are irregularly rugose and pitted. The basal surfaces are flat and have a distinctive pattern representing the ISFB making up the primary tissue of the ossicles.

**Figure 3:**
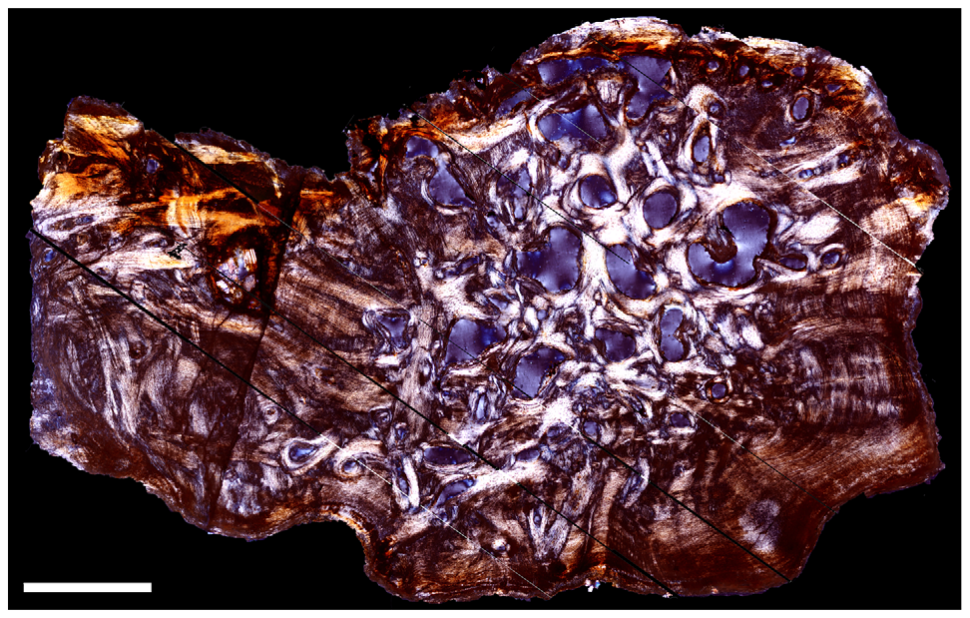
Dunvegan ossicle histology. Composite mosaic image of thin section through ossicle of TMP 2012.054.0002 in cross-polarized light. Orientation uncertain. Scale bar equals 1.0 mm.

**Figure 4:**
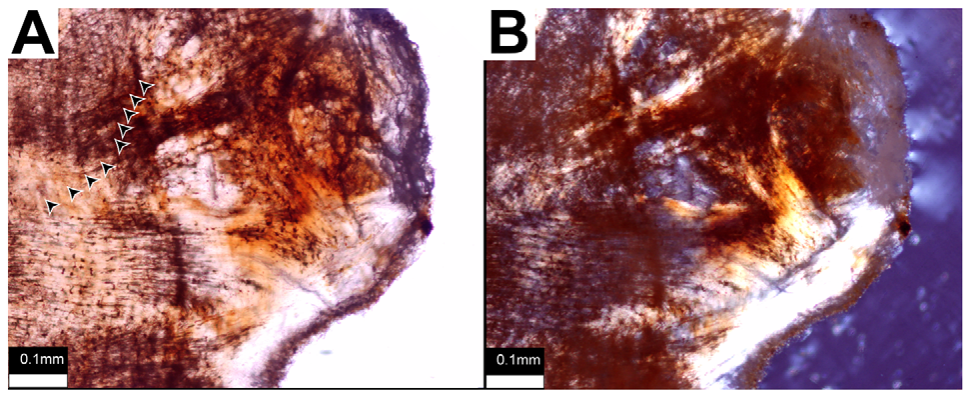
Primary ossicle tissues. Details of primary tissue in ossicle of TMP 2012.054.0002. A) Plane-polarized light showing zonation (growth marks indicated by arrowheads). B) Cross-polarized light showing ISFB.

**Figure 5:**
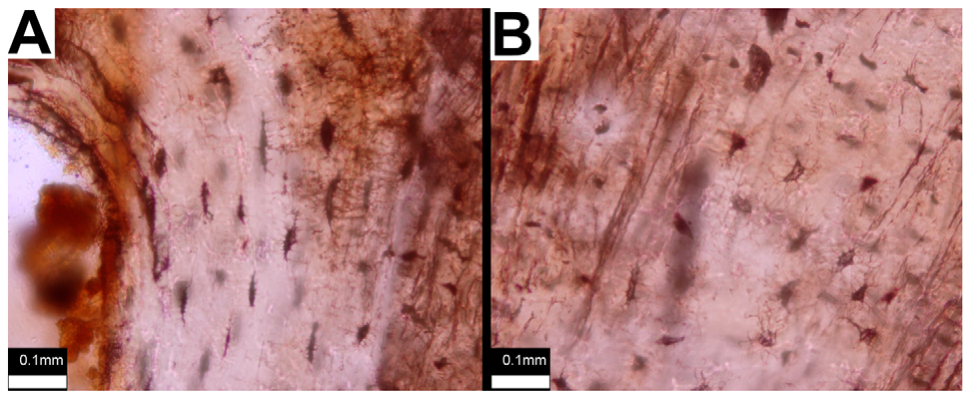
Ossicle osteocyte lacunae. Comparison of osteocyte lacuna morphology in primary (A) and secondary tissue (B) in an ossicle of TMP 2012.054.0002. Orientation uncertain. Scale bar equals 0.10 mm.

The primary tissue of the sectioned ossicle from TMP 2012.054.0002 (Fig. 3) is composed entirely of ISFB. Near the margins, zonation is overprints the pattern of structural fibers (Fig. 4). The lines of zonation, however, are not as distinct enough to be confidently called lines of arrested growth because they do not show a discreet hypermineralized line indicating a cessation of element growth. The ISFB are not as highly organized in terms of arrangement as those reported for the osteoderms of derived nodosaurids or the ossicles of sauropods [22]–[23], [27]–[28]. This is similar, however, to the condition reported for the ossicles of *Edmontonia* and *Euoplocephalus* [22] and unlike the radial pattern described for the ossicles of the basal ankylosaur *Antarctopelta* [29]. Secondary tissue comprises almost 50% of the cross sectional area of the ossicle and consists of trabecular bone. Osteocyte lacunae in the primary tissue, unlike the elongate lenticular lacunae of the secondary lamellar bone, are irregularly shaped (Fig. 5). Lacunae in both the primary and secondary tissue have canalliculi.

## Discussion

Other dinosaurian groups known to have had ossicles (namely stegosaurs and sauropods) are unknown in Late Cretaceous Canadian sediments. To date, secondary remodeling is not known to occur in sauropod ossicles and their orthogonal pattern of ISFB is stronger than in ankylosaurs [22]–[27]. Although stegosaurs cannot be ruled out on the basis of macro/microstructure, because their ossicle/gular osteoderm histology is unknown at present, the occurrence of the Dunvegan ossicles in the Upper Cretaceous makes them less parsimonious candidates than ankylosaurs.

The best possible alternative candidate for the taxonomic identification of the Dunvegan is crocodilian. Crocodilian osteoderms are common as fossils from Upper Cretaceous sediments in western Canada. Like some ankylosaur osteoderms/ossicles, the basal surface has a fine cross-hatch pattern corresponding to the connective tissue fascia that separates the element from epaxial musculature. The external surface, however, is characterized by distinctive sculpturing composed of numerous round pits and grooves that radiate from a midline keel [22]. This is a noticeable difference between crocodilian and ankylosaur osteoderms. In addition, crocodilian osteoderms largely develop in the loose, superficial dermis, leading to less dense structural fibers in the primary mineralized tissue [30]–[34]. Ankylosaur osteoderms/ossicles show dense ISFB networks, suggesting a greater contribution from the dense dermis [22]. Therefore, the external morphology and histology of the Dunvegan ossicles indicate that they are from an ankylosaur.

Unlike larger osteoderms, ossicles show no consistent differences among taxa, at least among derived nodosaurids and ankylosaurids [22]. Therefore, the ossicles sampled here cannot be identified to any particular ankylosaur group. Zonation in the form of annuli in modern crocodilians has been strongly correlated with age [35]; however, this association has not been tested for ankylosaur osteoderms or ossicles. Ankylosaurs likely had a delayed onset of osteoderm mineralization, more so than modern crocodilians, and similar to the heterochronic condition reported for stegosaur osteoderm mineralization [22],[28],[36]. The zonation observed in TMP 2012.054.0002 may be annual, but the growth marks are not continuous around the entire circumference of the ossicle and, in places, have been reabsorbed by secondary remodeling. The utility of growth marks in ankylosaur ossicles will need to be tested against another form of age determination (i.e., postcranial long bone histology).

Although the Dunvegan Formation has been previously known to contain abundant shark teeth from other localities near the Dunvegan Bridge on the Peace River [37], as well as an articulated, well-preserved fish from a fortuitous subsurface encounter [15], this is the first published record of dinosaur skeletal remains from the formation. Further west in Alberta and into British Columbia, dinosaur ichnites have previously been recorded from the Dunvegan Formation, including trackways of ankylosaurs [14],[38]. This discovery of skeletal fossils opens the possibility that further finds may be able to link some of these trackways with their potential trackmakers more closely.

Finally, the spatial location of the fossils is interesting from a biogeographic perspective. This locality represents the most northerly occurrence of ankylosaur skeletal remains during the Cenomanian, and further solidifies this group, and dinosaurs in general, as persistent residents of high latitude regions [39]–[41]. The region experienced large fluctuations in solar radiation through the year due to its high latitude, likely leading to seasonal growth patterns in vegetation, however these dinosaurs likely persisted in the area as they would have been physically unable to migrate long distances [15],[41]–[42]. Although temperatures would have been warmer and more equable at the time [19], they would have nevertheless experienced at least somewhat cooler winter temperatures.

## Conclusions

Although the ankylosaur remains from the Dunvegan Formation are generically indeterminate, their unique geographic and temporal position makes them an important data point for dinosaur biogeography. As well, the presence of dinosaurian remains from pre-Santonian deposits in Alberta suggests that, with further effort in many of these under-examined areas, there is the potential for additional finds in the region. High latitude dinosaur-bearing deposits are more poorly known in general than mid-latitude regions, although this is not due to the animals not being present, but more likely a function of search intensity.
